# Exploring Capabilities of Large Language Models such as ChatGPT in Radiation Oncology

**DOI:** 10.1016/j.adro.2023.101400

**Published:** 2023-11-04

**Authors:** Fabio Dennstädt, Janna Hastings, Paul Martin Putora, Erwin Vu, Galina F. Fischer, Krisztian Süveg, Markus Glatzer, Elena Riggenbach, Hông-Linh Hà, Nikola Cihoric

**Affiliations:** a Department of Radiation Oncology, Kantonsspital St. Gallen, St. Gallen, Switzerland; b School of Medicine, University of St. Gallen, St. Gallen, Switzerland; c Institute for Implementation Science in Health Care, University of Zurich, Zurich, Switzerland; d Department of Radiation Oncology, Inselspital, Bern University Hospital and University of Bern, Switzerland

## Abstract

**Purpose:**

Technological progress of machine learning and natural language processing has led to the development of large language models (LLMs), capable of producing well-formed text responses and providing natural language access to knowledge. Modern conversational LLMs such as ChatGPT have shown remarkable capabilities across a variety of fields, including medicine. These models may assess even highly specialized medical knowledge within specific disciplines, such as radiation therapy. We conducted an exploratory study to examine the capabilities of ChatGPT to answer questions in radiation therapy.

**Methods and Materials:**

A set of multiple-choice questions about clinical, physics, and biology general knowledge in radiation oncology as well as a set of open-ended questions were created. These were given as prompts to the LLM ChatGPT, and the answers were collected and analyzed. For the multiple-choice questions, it was checked how many of the answers of the model could be clearly assigned to one of the allowed multiple-choice-answers, and the proportion of correct answers was determined. For the open-ended questions, independent blinded radiation oncologists evaluated the quality of the answers regarding correctness and usefulness on a 5-point Likert scale. Furthermore, the evaluators were asked to provide suggestions for improving the quality of the answers.

**Results:**

For 70 multiple-choice questions, ChatGPT gave valid answers in 66 cases (94.3%). In 60.61% of the valid answers, the selected answer was correct (50.0% of clinical questions, 78.6% of physics questions, and 58.3% of biology questions). For 25 open-ended questions, 12 answers of ChatGPT were considered as “acceptable,” “good,” or “very good” regarding both correctness and helpfulness by all 6 participating radiation oncologists. Overall, the answers were considered “very good” in 29.3% and 28%, “good” in 28% and 29.3%, “acceptable” in 19.3% and 19.3%, “bad” in 9.3% and 9.3%, and “very bad” in 14% and 14% regarding correctness/helpfulness.

**Conclusions:**

Modern conversational LLMs such as ChatGPT can provide satisfying answers to many relevant questions in radiation therapy. As they still fall short of consistently providing correct information, it is problematic to use them for obtaining medical information. As LLMs will further improve in the future, they are expected to have an increasing impact not only on general society, but also on clinical practice, including radiation oncology.

## Introduction

Advancements in natural language processing (NLP) have led to the development of language models that are able to process large amounts of textual data. These recently developed large language models (LLMs) have shown remarkable capabilities in the interpretation of text and in the generation of well-formed text. Some of the most powerful models used today are based on the generative pretrained transformer 3/3.5/4 (GPT-3/GPT-3.5/GPT-4) models, developed by OpenAI. ChatGPT, an LLM with 175 billion parameters based on GPT-3.5 and further training through extensive human feedback,^1^ has achieved impressive results in different subjects and tasks that usually require profound knowledge and extensive understanding and reasoning for humans to perform.^2^^,^^3^ Since its release in November 2022, it has gained a lot of attention both publicly and scientifically, due to its good performance and wide knowledge in a variety of fields.

With the rapid technological advancements of LLMs and the newly arisen capabilities they show, it is very likely that these will have major implications for health care,^4^ particularly as the technology continues to evolve. However, at the current time, still relatively little is known about what the new generation of LLMs may be used for in the clinical environment, not only in the context of general medical question-answering but also in clinical routine and in specialized medical fields. Artificial intelligence (AI) and NLP may be of particular interest in radiation oncology, due to it being a very specialized technical and data-driven medical discipline that requires very domain-specific expertise beyond general medical knowledge.

To explore the current capabilities of modern conversational LLMs in radiation therapy, the International Society for Radiation Oncology Informatics performed this study to descriptively evaluate the capabilities of ChatGPT in answering domain-specific questions related to radiation oncology.

## Methods and Materials

### Study design

The objective of the study was to explore how well ChatGPT (GPT-3.5-turbo) was able to answer relevant clinical questions as well as more basic general knowledge questions about radiation oncology. For this purpose, a 2-part evaluation approach was used.

### Part 1: Evaluation of multiple-choice questions about general knowledge in radiation oncology

To investigate the broader “general knowledge” of ChatGPT in radiation oncology, a test consisting of multiple-choice questions (with 4 A-D answers per question) was created. The questions were considered to be easily understandable, with only one unambiguously defined answer being clearly correct. The questions also contained the instruction “Provide only the correct letter (A, B, C, or D) as answer” for the LLM to select 1 of the answers. Questions were grouped into the 3 thematic groups: clinical, physics, and biology. A total of 70 questions, each with 4 possible answers, were created upon agreement by the 3 study coordinators FD, PMP and NC. The questions were considered suitable (meaning clearly understandable and unambiguously answerable) by all 3 physicians. Thematically, 44 questions were clinical questions, 14 questions were physics questions, and 12 questions were biology questions.

The questions were posed to ChatGPT via the web interface provided by OpenAI.^5^ To reduce bias due to the retention of previous questions and answers, a new chat session was started for each question. The text was entered in the English language and no adaptations were made to the answers provided by ChatGPT.

### Part 2: Physician-based evaluation of answers to relevant clinical questions

Radiation oncology is a complex medical field with many factors to consider and many uncertainties in clinical decision-making. As a result, many relevant clinical questions often do not just have “one clear answer” that can definitely be identified as either “correct” or “incorrect”. Therefore, to evaluate some of these more complex relevant questions, we used a physician-based evaluation approach in the second part of the study.

With the objective of evaluating the answers of ChatGPT to open-ended questions relevant for radiation therapy, a list of text-based questions/tasks (without multiple-choice answers) was created. Open-ended questions/tasks (intended to cover different aspects of radiation therapy) were created by the 3 study coordinators. After several adaptations and revisions regarding content and formulation, the 3 clinicians agreed upon a list of 25 questions/tasks that were considered relevant for radiation therapy and adequate for the study. The questions were grouped into the topics “Patient evaluation/indication”, “Treatment planning”, “Plan evaluation”, “Treatment and side effects”, and “Others”.

Physicians from the radiation oncology departments of the Cantonal Hospital of St. Gallen and of the University Hospital of Bern were asked to evaluate the quality of the answer to each question using an evaluation form. To reduce bias, the evaluators were not informed about the whole study design and were not told that the answers were given by AI. The evaluating physicians just received the study documents with the request to evaluate the quality of answers to medical questions.

The two main relevant factors defining the overall quality of an answer were correctness and usefulness. While the correctness and usefulness of an answer clearly correlate, they are not necessarily identical (e.g., the question “Why should a patient with breast cancer receive adjuvant radiation therapy?” could be answered with “To treat the disease”. In such a case, the answer would be correct but not very useful). To address this, the physicians doing the evaluation were asked to separately evaluate the quality of an answer regarding correctness and regarding usefulness. A 5-point Likert scale (1 representing “very bad”; 2 “bad”; 3 “acceptable”; 4 “good”; 5 “very good”) was used for the evaluation.

Because there may be disagreements due to limited medical knowledge about individual circumstances, there is not always one clear answer to a given open-ended question. Therefore, the radiation oncologists were asked to do the evaluation based on generally accepted medical knowledge and not to insist on personal beliefs and opinions.

Furthermore, the physicians were asked whether adaptations to the answers should be made to improve their quality and to provide comments about how to do so. The physicians were allowed to search medical literature to check on the scientific background of a specific question.

Seven radiation oncologists at the radiation therapy departments of the Cantonal Hospital of St. Gallen and of the University Hospital of Bern were contacted for participation in the study without being told that the answers were given by AI. Six physicians agreed to participate and returned their completed evaluation forms. The participating radiation oncologists had a median of 6.5 years of clinical experience in radiation oncology (range, 1.5-10 years).

**Figure 1:**
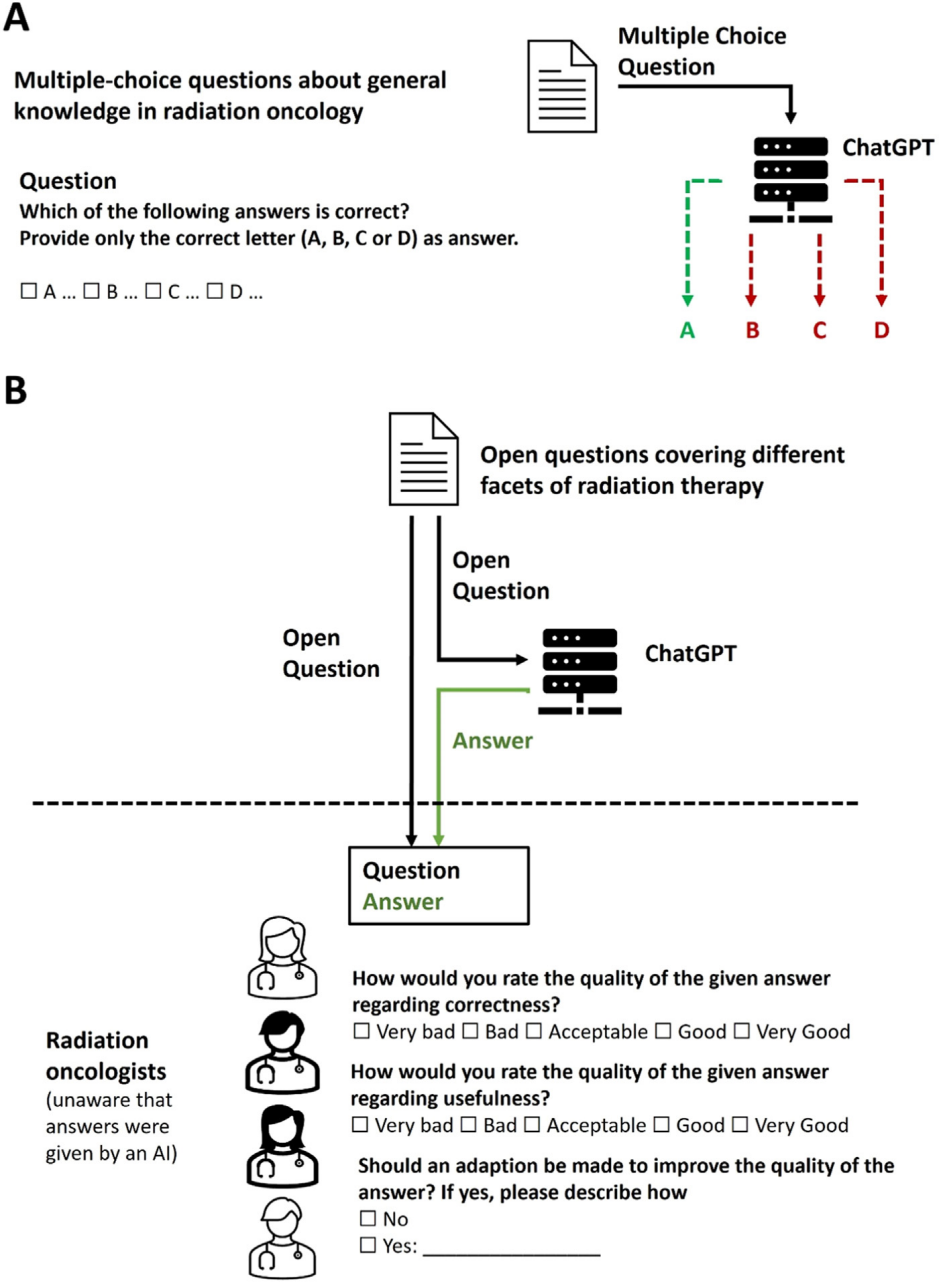
Schematic illustration of the study design. (A) Multiple-choice questions with one correct answer choice were answered by ChatGPT. The portion of valid and of correct answers was determined. (B) Open-ended questions/tasks of radiation therapy were answered by ChatGPT. The answers were then evaluated by independent radiation oncologists. To avoid a possible bias, the physicians were not informed that the answers were given by artificial intelligence.

The overall study design (parts 1 and 2) is illustrated in Fig. 1.

### Ethical considerations

No approval from an ethics committee was required for this study. A declaration of nonresponsibility was issued by the local ethics committee of Eastern Switzerland.

### Data and statistical analysis

After collection of the answers to the multiple-choice questions for part 1 of the study, each answer was evaluated to determine whether a clear assignment to one of the provided answers (A-D) was possible. Answers of ChatGPT that failed to select one of the four provided answers were defined as invalid. The portion of valid, correct, and incorrect answers was determined for all questions, as well as for each of the clinical, physics, and biology questions.

For part 2 of the study, the performance of ChatGPT was examined based on physicians’ evaluation form ratings. The values on the 5-point-Likert scale ranging from 1 (“very bad”) to 5 (“very good”) were used to obtain a score for the quality of an answer regarding correctness and usefulness. The overall score for ChatGPT on individual questions was calculated as the mean of the values given by the individual raters.

The evaluation of the individual radiation oncologists as well as interrater agreement (IRA) were determined. IRA on individual questions/tasks was determined by calculating r_WG_, and overall agreement on all items was determined by calculating r_WG_ (J).^6^ Furthermore, the intraclass correlation coefficient (ICC) was calculated with a 2-way mixed model with absolute agreement^7^. R_WG_, r_WG_ (J), and ICC can have values between 0 and 1, with low values indicating a low level of agreement and values close to 1 indicating a high level of agreement. Statistical analyses were performed using SPSS 29.0.0.0 and Microsoft Excel.

The comments given by the physicians to improve the quality of the answers were examined by content analysis. Each comment was assigned to 1 or several of the following 3 categories: “comment mentioning errors or inaccuracies in the answer”, “comment recommending further details or clarification to the answer”, and “comment not directly related to the quality of the answer”. The frequencies of these categories were determined.

## Results

### Performance of ChatGPT in answering multiple-choice questions

For 66 of the 70 answers (94.3%) given by ChatGPT to the multiple-choice questions, a clear assignment to 1 of the 4 provided answers was possible. For the other 4 questions, the LLM did not select one of the answers but provided the information that it was an AI language model with knowledge cutoff at September 2021 and that it was unable to answer the question. As all the questions could in fact be answered with knowledge available before September 2021, these 4 answers were deemed invalid.

**Figure 2:**
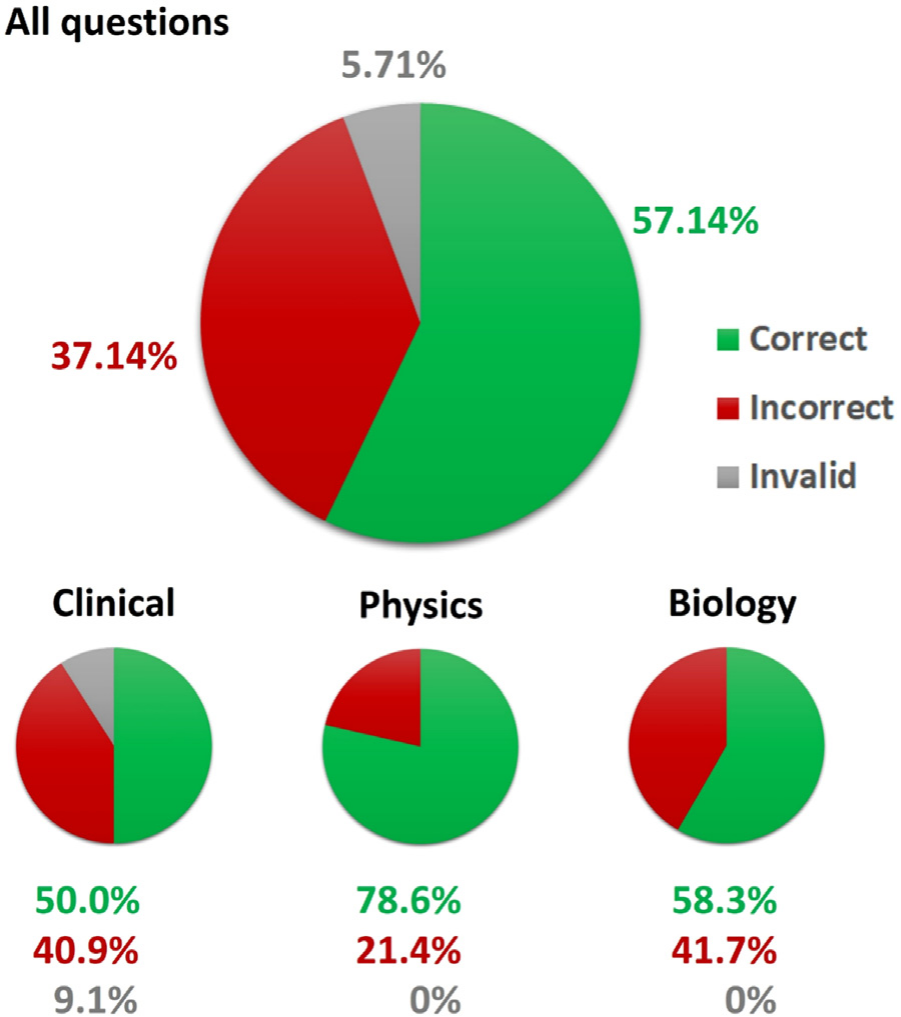
Portion of correct, incorrect, and invalid answers of ChatGPT to the multiple-choice questions.

For 40 questions, ChatGPT selected the correct answer (57.14% of all questions, 60.61% of validly answered questions). Regarding the thematic subgroups, 22 of the 44 clinical questions (50%), 11 of the 14 physics questions (78.57%), and 7 of the 12 biology questions (58.33%) were answered correctly (Fig. 2). All the questions, together with the answers from ChatGPT, are provided in Appendix E1.

### Performance of ChatGPT in answering open-ended questions as evaluated by physicians

Out of the total of 6 × 25 = 150 open-ended question evaluations, the correctness of the answers given by ChatGPT was “very good” 44 times (29.3%), “good” 42 times (28%), “acceptable” 29 times (19.3%), “bad” 14 times (9.3%), and “very bad” 21 times (14%). Mean scores ranged from 1.50 to 5.00 (mean of all scores 3.49; median of all scores 3.67).

**Figure 3:**
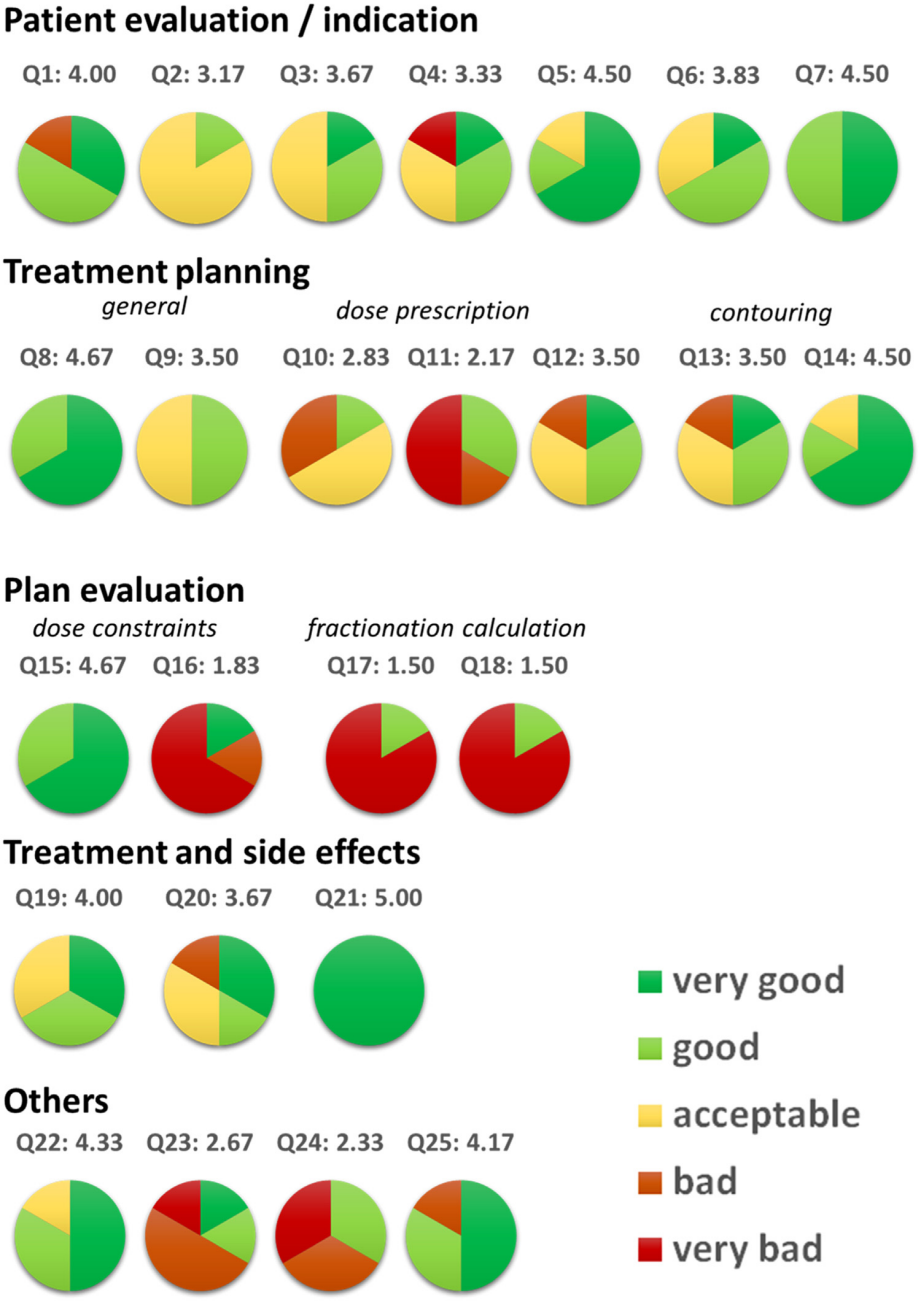
Evaluation results regarding the correctness of the answers given by ChatGPT. Score values of each answer were calculated as the mean of individual score values given by the radiation oncologists.

The correctness of 13 answers was considered “very bad” or “bad” by at least one of the evaluators, leaving 12 answers that were considered “acceptable”, “good”, or “very good” by all physicians. Four answers were concordantly considered “good” or “very good”, with one answer reaching a perfect result, concordantly rated as “very good”. Results for correctness are presented in Fig. 3.

Slightly different but similar results were obtained for the usefulness of the answers: “very good” 42 times (28%), “good” 44 times (29.3%), “acceptable” 29 times (19.3%), “bad” 14 times (9.3%), and “very bad” 21 times (14%). Fifteen answers were deemed “bad” or “very bad” by at least one of the physicians. The same 4 answers that were considered “good” or “very good” regarding correctness were also concordantly deemed “good” or “very good” regarding usefulness. Mean scores ranged from 1.50 to 5.00 (mean of all scores, 3.48; median of all scores, 3.5). Individual results for usefulness are presented in Appendix E3.

### Comments for improving the quality of answers

For 24 questions (96%), at least one of the radiation oncologists provided a comment to improve the quality of the answer. Overall, comments were provided in 75 of 150 cases. Forty comments addressed some kind of error or inaccuracy of an answer. Forty-one comments recommended adding further details or clarification to an answer to improve its quality. Four comments did not directly relate to the quality of the answer. A comment addressing an error/inaccuracy was made in 16 answers (64%), while a recommendation to add further details was given in 20 answers (80%) by at least one of the radiation oncologists. The results of the classification of the comments on individual questions or tasks are presented in Appendix E4.

### Evaluations of individual physicians and interrater agreement

**Figure 4:**
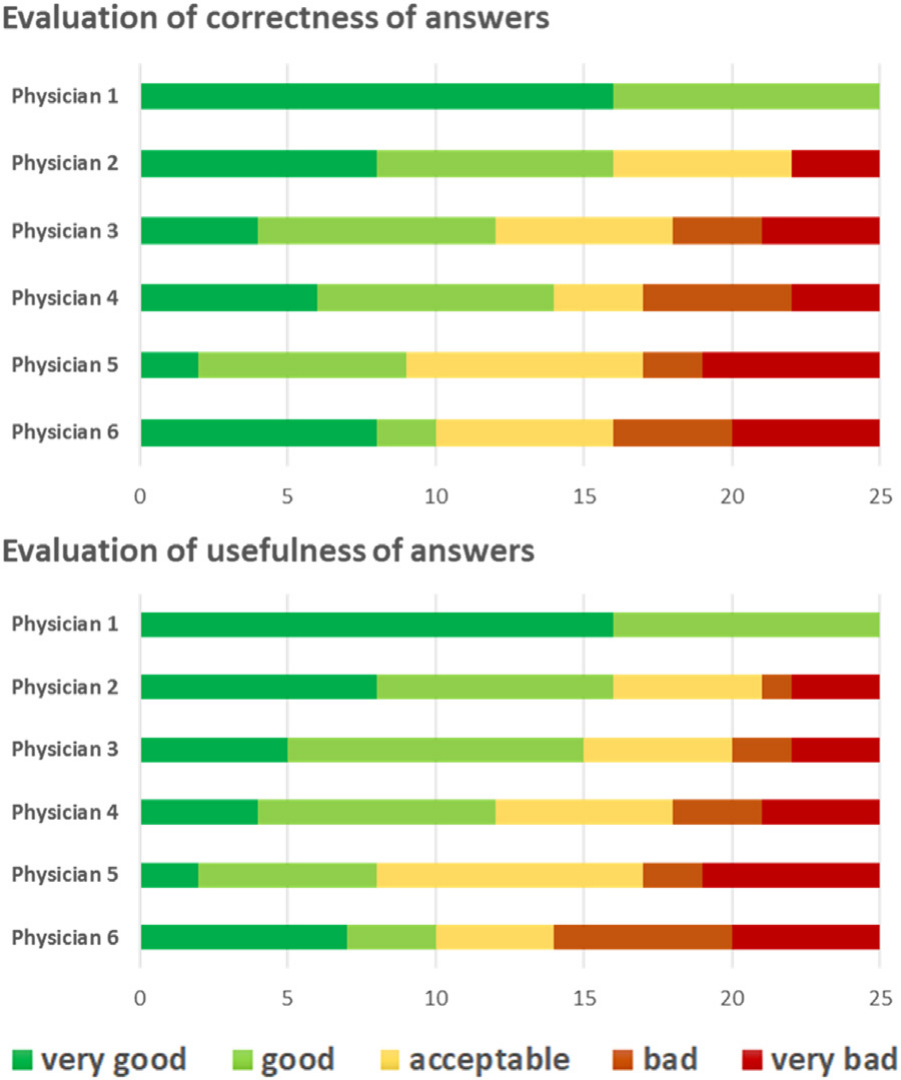
Evaluation of correctness and usefulness by individual radiation oncologists.

The evaluations of the 6 physicians varied (Fig. 4). An answer was considered “very bad” or “bad” regarding correctness and/or usefulness in 0, 4, 7, 8, 8, and 11 cases (median 7.5) by the individual physicians.

IRA on the evaluation of individual questions/tasks was determined by calculation of r_WG_, which yielded results ranging from 0.00 to 1.00. The r_WG_ values had a median of 0.6 (correctness) and 0.45 (usefulness). IRA was low, with r_WG_ values of 0.00 for 3 of the 25 questions regarding both correctness and usefulness (questions 11, 16, and 23).

Overall agreement was determined by calculation of r_WG_(J), which had a value of 0.959 for correctness and 0.956 for usefulness. Results for the individual questions/tasks are presented in Appendix E4. The additionally calculated ICC had a value of 0.892 (95% CI, 0.810-0.946) for correctness and 0.886 (95% CI, 0.799-0.943) for usefulness.

### Answers of ChatGPT to individual questions

Some individual answers given by ChatGPT to the open-ended questions are presented later in this article. The total list of open-ended questions and answers is provided in Appendix E2.

### Examples of “acceptable/good” answers

One exemplary question from the group “Patient evaluation / indication” is question 3: “What are the indications for adjuvant axillary radiotherapy in a patient with breast cancer?” The answer given by ChatGPT was:

*Adjuvant axillary radiotherapy is typically indicated in breast cancer patients with positive axillary lymph nodes, as a means to reduce the risk of local regional recurrence. This treatment is usually offered in conjunction with other adjuvant therapies such as systemic therapy and surgery. The decision to offer adjuvant axillary radiotherapy is based on several factors including the number of positive axillary lymph nodes, the size of the primary tumor, and other patient-specific factors.*

Regarding correctness, the answer was evaluated as “very good” by one physician, as “good” by 2 physicians, and as “acceptable” by 3 physicians. It yielded a score of 3.67 regarding correctness and of 3.50 regarding usefulness (Fig. 3; Appendix E3).

As mentioned, for one question, ChatGPT yielded a perfect result, with all physicians considering it “very good” (score of 5.0 for both correctness and usefulness). The corresponding question is question 21: “A patient with limited disease small-cell lung cancer had chemotherapy with concurrent radiotherapy for 6 weeks. After 4 weeks of the treatment, he is beginning to develop symptoms of pain during swallowing (VAS 6/10), which he describes as burning. He also suffers from impaired swallowing, which is why he reduced eating. What is the most likely reason for the symptoms and how could you help the patient?”

Answer: *The most likely reason for the patient's symptoms of pain during swallowing and impaired swallowing is radiation-induced esophagitis, which is a common side effect of concurrent chemoradiotherapy for lung cancer. The symptoms can be due to inflammation and injury of the esophageal mucosa caused by the radiation.*

*To help the patient, the following measures can be taken:*

1. *Pain management: Over-the-counter pain relievers or prescription pain medication can be used to manage the pain.*
2. *Diet modification: Soft, bland foods and frequent small meals can help alleviate symptoms and improve swallowing. Avoiding spicy and acidic foods can also help.*
3. *Mouth care: Good oral hygiene, such as regular mouth rinses and gargles, can help to soothe the throat and prevent infections.*
4. *Lubricating agents: Using agents such as honey, glycerin, or saliva substitutes can help to lubricate the throat and improve swallowing.*
5. *It is important to consult a medical professional for proper evaluation and management of the patient's symptoms, as the above measures may not be sufficient to alleviate symptoms in all cases. Additionally, if the symptoms persist or worsen, further investigations such as endoscopy may be necessary to rule out other causes.*

### Example of a “bad” answer

While these two examples show that some of the answers of ChatGPT are of acceptable or good quality, the answers to some other questions were of poor quality. The two answers with the worst results were about fractionation calculations using the linear-quadratic (LQ) model. One example is question 17: “What is 20 × 3 Gy in EQD2 based on the linear-quadratic model using an α/β= 2?”

Answer: *The equivalent dose in 2 Gy fractions (EQD2) can be calculated using the linear-quadratic model and an α/β of 2 as follows:*

EQD2=20×3Gy*(2+(2/α/β))=20×3Gy*(2+(2/2))=20×3Gy*4=240Gy

*So, the EQD2 for 20 fractions of 3 Gy each with an α/β of 2 is 240 Gy.*

In this case, the answer of ChatGPT can clearly be defined as incorrect (the formula used by ChatGPT is incorrect, and the correct answer would be 75 Gy).^8^ The answer was considered as “very bad” for both correctness and usefulness by 5 of the 6 radiation oncologists.

## Discussion

We aimed to explore the capabilities of ChatGPT in answering questions in the context of radiation therapy. We showed that some of the answers given by the model may be of good or very good quality.

ChatGPT has been tested across a wide range of subjects and has been shown to achieve success in fields such as business management^9^ and law school examinations.^10^ When applied within medicine, ChatGPT has shown remarkable results in medical question answering and performed comparably to the level of a third-year medical student.^11^ Furthermore, ChatGPT performed near the level of the passing threshold on the United States Medical Licensing Examination in a study by Kung et al.^12^ In another study by Ayers et al, evaluators preferred the responses of ChatGPT over physician responses to patient questions from a social media forum in 78.6% of cases.^13^

Our findings show that ChatGPT may also provide some helpful and correct answers in radiation therapy, with an anticipated success rate of about 50% to 70% correct answers in a multiple-choice test like the one used in our study. For the more complex treatment-related questions, under the evaluation criterion that the correctness of given answers should be deemed “acceptable” or better by all clinicians in our physician-based evaluation, the model would have fulfilled this requirement in 12 out of 25 questions (48%).

With the continuing progress in the field of LLMs and the fine-tuning of models or application of other optimization techniques, the performance of future LLMs is likely to be considerably improved.

To avoid false and possibly harmful answers, models may also be adapted to behave in a more cautious way, like giving medical answers only if well-established medical knowledge exists. To overcome such problems, current research focuses on combining models with explicit knowledge bases.^14^

### LLMs in medicine

While models such as ChatGPT can provide some correct and useful answers in radiation therapy, they are in principle rather general models without special optimization for the medical domain.^15^ Other models have been developed specifically for application in medicine. One of the most powerful models is Med-PaLM, developed by Google.^16^ As reported by the researchers involved in the development of Med-PaLM, it can provide helpful answers, often near the level of clinicians. The newer generation, Med-PaLM 2, was shown to answer medical examination questions at an “expert doctor level.” It reached an accuracy of 85% on US medical licensing style questions, outperforming its predecessor by 18%.^17^ An important thing to keep in mind is the rapid pace of progress (e.g., the results of Med-PaLM were published in December 2022; the announcement of Med-PALM 2 results was just 4 months later, in March 2023). However, because Med-PaLM is not available to the public, we were not able to use it in our study.

The recent advancements of LLMs offer immense possibilities for application in medicine. With the advancements in AI continuing, it is likely that new AI technologies will profoundly change health care.^18^^,^^19^ LLMs offer language capabilities, a key feature for processing data based on domain-specific knowledge, which will be essential for future applications in medicine. The multimodal capabilities of the newest models, such as those combining images and text, unlock an even wider set of possibilities for processing medically relevant data in sophisticated ways.

LLMs also have the potential to assist physicians in their daily clinical lives. For example, LLMs could be very helpful in administrative work. In a pilot study by Ali et al, ChatGPT wrote patients clinical letters with high scores regarding factual correctness and humanness, as evaluated by physicians.^20^

Further use of LLMs may include medical education,^12^ research,^21^ or application in clinical-decision support systems.^22^ For now, it remains unclear how recent advancements will impact general society and medicine. LLMs have begun to be used for medical advice with unknown consequences.^4^ With the fast progress in the field, models such as Chat-GPT, MedPaLM, or GPT-4 are just the predecessors of models that may be much more powerful and may considerably impact clinical practice^23^.

### LLMs in radiation therapy

NLP and LLMs may be of particular interest in radiation therapy.^24^ In general oncology as well as radiation oncology, physicians are faced with complex medical situations with many individual factors. Profound medical knowledge—which frequently changes due to new therapeutic options and new findings from clinical trials—is essential to making adequate decisions. With a lot of uncertainty and limited knowledge in individual oncological situations, AI-based support of clinical decision-making is of high interest.^25^ Furthermore, radiation oncology is in part a very technical and data-driven discipline, characterized by a high level of data processing.^26^ Radiation oncology information systems (ROCIS) are broadly used to manage data about patient treatment schedules, treatment plans, treatment delivery, and documentation.^27^^,^^28^ Many steps involved in radiation therapy can be assessed and supported using IT and AI systems. The application of ROCIS facilitates direct workflow integration of such systems in clinical care. This allows implementation of AI solutions not only for circumscribed tasks within radiation therapy, but more generally in multidisciplinary oncological situations (e.g., application of AI systems in the multidisciplinary treatment of prostate cancer).^29^ LLMs may play a key role in the future design of comprehensive oncological data systems. If the models can be coupled appropriately with medical evidence, LLMs might indeed be highly valuable for radiation therapy.^30^ Our study shows that modern LLMs have the potential to provide useful answers not only regarding general subjects, but also in highly specialized topics of radiation therapy.

### Problems and drawbacks

Despite the impressive capabilities of the new LLMs, it has been repeatedly shown that they have considerable limitations, so their output needs to be interpreted with great caution.^31^

One of the major issues with using advanced conversational models as sources of medical advice is that they may “hallucinate”, meaning that an LLM may generate text with illusory statements not based on correct data.^32^ An answer given by an LLM consists of a sequence of words that is the result of statistical calculations. Which sequence of words is created depends on its probability, as determined during the training of the model. Sequences of text that occur more commonly in training data are assigned higher probabilities during the foundational training phase of the model, and sequences of text that are formulated in a suitable way within a dialogue context are assigned higher probabilities during the instruction training phase of the model. How well a model can answer domain-specific questions therefore depends on the design, training data, and size of the model. However, the model is not directly coupled to evidence but rather represents a synthesis of its training data with generative capability. Therefore, there is no constraint preventing it from generating incorrect statements that appear as if they were evidence-based. In oncology and medicine in general, this obviously presents a considerable problem regarding the safety and application of such a model. As mentioned, the combination of language models with explicit knowledge bases is a promising future direction to enable overcoming a part of this problem.^14^

Furthermore, one should be aware that LLMs are not equally powerful in different tasks and still have partly limited capacities. As an example, in our study, ChatGPT failed to consistently answer questions requiring fractionation calculation. It has been shown that LLMs have limited performance when solving arithmetic reasoning and calculation tasks.^33^ Unlike natural language understanding, calculations typically have a single correct answer, making the task of generating accurate solutions more challenging. Moreover, they require specific abstraction and reasoning skills that are not well supported by the architecture and training of language models.

Another problem arises from the fact that every model is dependent on the data it was trained on. This can lead to wrong and biased results (such as biases related to sex, gender or ethnicity), as LLMs may adopt unwanted features from their training data.^34^^,^^35^

Despite some good results, ChatGPT failed to consistently provide correct and good answers for many of the questions in our study. Since the consequences of wrong advice can be severe in medicine, the quality bar for clinical application of such technologies is very high, which is why LLMs in their current form should not be used directly for clinical decision-making, although they may provide supplementary language-related functionality in larger decision-making applications. While LLMs will improve and will likely play an important role in future health care, they will likely always have limitations that users should be aware of. In any case, LLMs cannot and should not be used to replace human doctors but to assist them in their work.^4^

### Usage of LLMs by patients

In the current state, it is not advisable to use LLMs when seeking medical advice. However, models such as ChatGPT have gained a lot of attention in recent months and are easily accessible. Furthermore, GPT-4 has been introduced into the Bing web search of Microsoft,^36^ and both Microsoft and Google have announced plans to further implement the new models into their software products. It is thus very likely that radiation oncologists and other clinicians will soon have consultations with patients who have previously consulted an LLM such as ChatGPT before attending the appointment with their treating physician. Clinicians should therefore be aware of the capabilities and limitations of these new technologies. While the support of radiation oncologists in daily clinical life by LLMs may not yet be a reality, LLMs will already have an impact on patients seeking information about their oncological situation.

### Study limitations

Our study has several limitations. In general, the evaluation of LLMs in medicine is challenging and currently a subject of open discussion.^16^ While the performance of LLMs such as Med-PaLM is assessed using benchmarks like medical question–answering data sets, this approach fails to encompass all relevant factors needed in daily clinical life. Furthermore, despite the vast amount of medical literature available, the best advice for an individual patient's situation is not always known. Many relevant questions in radiation oncology do not have one defined correct answer, but an answer may be of higher or lower quality. In our study, we used a set of multiple-choice questions about basic knowledge as well as a physician-based evaluation to assess the quality of answers given by ChatGPT. However, the physician-based evaluation is prone to the personal beliefs and subjective factors of the clinicians and may fail to obtain an objective assessment. As we have also seen by comparing the evaluations of the different participating physicians, the interrater agreement for some questions/tasks was quite poor. Even though we saw an overall high level of agreement, a consensus on individual answers may not always be reached. This limits the possibility of assessing the quality of a given answer in some situations.

Furthermore, our study used a limited number of 70 multiple-choice questions and 25 open-ended questions. While the questions were created with the intention of covering different facets of radiation therapy, our study does not provide a comprehensive or systematic evaluation of LLMs in radiation therapy. Overall, the study can only be of descriptive nature, and the results do not allow further generalization.

In future work, testing the performance of LLMs in a more systematic way would ideally encompass a larger set of questions/tasks, evaluation by many physicians comparing different models and prompting techniques, as well as comparing it to the performance of clinicians and medical trainees. Furthermore, it should be noted that the development of benchmarks to evaluate the performance of LLMs is additionally challenging due to the lack of transparency about the training data used in model development. Ideally, models should be evaluated on their performance on questions that they have not seen in their training. Very complex and effortful systematic studies will be necessary to evaluate the role of LLMs in the clinical practice of future health care. This is beyond the scope of the current study, which was initiated by the International Society for Radiation Oncology Informatics to initially assess the capabilities of these new technologies in radiation oncology.

## Conclusion

We have shown that ChatGPT can provide correct and useful answers to some questions that are relevant in radiation therapy. However, because such models are currently not reliable and may lead to inaccurate or wrong answers, their output should be taken with caution. Nevertheless, clinicians should be aware of the capabilities and problems of LLMs, as patients may use them to seek medical advice. As the technology continues to evolve rapidly, LLMs are anticipated to have a major effect on the practice and future of medicine and radiation oncology.

## Disclosures

Nikola Cihoric is a technical lead for the SmartOncology project and medical advisor for Wemedoo AG, Steinhausen AG, Switzerland.

## Appendix Supplementary materials

Appendix1 - Multiple-choice questions Image, application 1

Appendix2 - Open-ended questions Image, application 2

Appendix3 - Additional Figures Image, application 3

Appendix4 - Additional Tables Image, application 4

## Footnotes

Supplementary material associated with this article can be found in the online version at doi:10.1016/j.adro.2023.101400.
