# Supplementary material for: Exploring Capabilities of Large Language Models such as ChatGPT in Radiation Oncology
Source: Adv Radiat Oncol. 2023 Nov 4;9(3):101400. doi: 10.1016/j.adro.2023.101400 (PMC10831180; doi:10.1016/j.adro.2023.101400)
Supplement: Appendix2 - Open-ended questions [file mmc2.docx]

The list of questions/tasks is intended to cover different aspects of radiation therapy. It includes some more general questions, some very specific questions to a presented clinical situation, some text-based tasks for fractionation calculation and summarization of a patient case, as well as questions to landmark trials important for radiation therapy.

Questions were used with the exact text as provided in this file. No further prompting was used.

A new session of ChatGPT was started for each question.

ChatGPT Jan30Version was used for the evaluation.

1. Please recommend the most appropriate therapy for low-grade glioma (2cm in diameter) diagnosed in the frontal lobe.

*Treatment for low-grade gliomas depends on several factors, including the location of the tumor, age, and overall health of the patient. The most common treatments for low-grade gliomas are surgery, radiation therapy, and chemotherapy.*

*In the case of a 2 cm diameter low-grade glioma in the frontal lobe, the most appropriate therapy would likely be a combination of surgery and radiation therapy. The goal of the surgery is to remove as much of the tumor as possible, and radiation therapy is used to help control the growth of any remaining cancer cells. In some cases, chemotherapy may also be used in conjunction with surgery and radiation.*

*It's important to note that the best treatment plan for an individual with a low-grade glioma will depend on their specific circumstances and should be discussed with a neuro-oncologist, who can provide more detailed information and guidance.*

2. What are the preferred treatment options for early-stage anal carcinoma?

*The preferred treatment options for early-stage anal carcinoma include:*

1. *Radiation therapy and chemotherapy: Radiation therapy can be used to shrink the tumor and may be combined with chemotherapy to increase its effectiveness.*
2. *Surgery: In some cases, surgical removal of the tumor may be recommended, especially if the cancer has not spread to other parts of the body.*
3. *Chemotherapy alone: In some cases, chemotherapy may be used as the primary treatment, especially if the patient is not a good candidate for radiation therapy or surgery.*

*The choice of treatment will depend on the size and location of the tumor, the patient's overall health, and other factors, and will be decided on a case-by-case basis by a team of healthcare professionals.*

3. What are the indications for adjuvant axillary radiotherapy in a patient with breast cancer?

*Adjuvant axillary radiotherapy is typically indicated in breast cancer patients with positive axillary lymph nodes, as a means to reduce the risk of local regional recurrence. This treatment is usually offered in conjunction with other adjuvant therapies such as systemic therapy and surgery. The decision to offer adjuvant axillary radiotherapy is based on several factors including the number of positive axillary lymph nodes, the size of the primary tumor, and other patient-specific factors.*

4. A patient is presented to the tumor board: Poor performance status (Karnofsky 60) and locally advanced non-small cell lung cancer (large cell carcinoma), G3, cT3 cNx cMx. Please suggest the most appropriate procedures for further clinical diagnosis and treatment.

*For the patient with locally advanced non-small cell lung cancer (large cell carcinoma) and poor performance status (Karnofsky 60), the following diagnostic and treatment procedures are recommended:*

1. *Computed Tomography (CT) scan of the chest and abdomen to assess the extent of the disease and look for any distant metastases.*
2. *PET/CT scan to confirm the diagnosis and evaluate for any distant metastases.*
3. *Bronchoscopy with biopsy to confirm the histology and subtype of the lung cancer.*
4. *Multidisciplinary tumor board review to discuss the best treatment options, taking into account the patient's performance status and overall health.*
5. *Palliative treatment such as chemotherapy or radiation therapy to relieve symptoms and improve quality of life.*
6. *In some cases, surgical resection may be considered if the patient is fit enough for the procedure and the cancer is surgically resectable.*
7. *Close monitoring and regular follow-up to assess response to treatment and adjust the treatment plan as needed.*

5. Is radiation indicated for early-stage, low-risk prostate cancer?

*The use of radiation therapy for early-stage, low-risk prostate cancer is a matter of debate and varies depending on various factors such as the patient's age, overall health, and preferences. Some doctors may recommend radiation therapy as part of a treatment plan, while others may recommend active surveillance or surgery. It is important to discuss the options with a medical professional to determine the best course of action.*

6. Ms. Smith is a 81 year old woman who has an unclear pulmonary nodule in the upper lobe of the left lung. The lesion has a spiculated morphology and is suspicious for malignancy. The FDG-PET-CT showed a moderate tracer uptake. There is no sign of lymph node involvement or a distant metastasis. In the bronchoscopy, no tumor cells were found in the bronchoalveolar lavage. Due to the localization of the lesion, no biopsy was possible. Ms. Smith has a known cardiac hypertension and an atrial fibrillation. What would be possible therapeutic options and advantages/disadvantages of each therapy?

*The therapeutic options for Ms. Smith's pulmonary nodule would depend on several factors such as the size, location and biopsy results of the nodule, and her overall health status. However, given the information available, some of the possible options are:*

1. *Observation: This option may be considered if the lesion is small and not growing, and the biopsy results are not conclusive. The advantage is avoiding the risks and side effects of invasive procedures or treatments.*
2. *Surgical Resection: If the lesion is biopsy proven to be malignant, then surgical resection would be the preferred option. The advantage is complete removal of the cancerous tissue, with the potential for cure. However, the disadvantages include the risks and side effects associated with surgery, as well as the need for general anesthesia and a long recovery time.*
3. *Radiation Therapy: This option may be considered if the lesion is not operable or if the patient is not a surgical candidate. The advantage is the ability to treat the lesion without surgery, but the disadvantages include the risk of radiation-induced damage to surrounding normal tissues, and the need for multiple treatment sessions over several weeks.*
4. *Stereotactic Body Radiation Therapy (SBRT): This is a type of high-precision, non-invasive radiation therapy that can deliver a high dose of radiation to a small target with minimal damage to surrounding tissues. The advantage is that it can be completed in a shorter period of time than conventional radiation therapy, but the disadvantages include the need for highly specialized equipment and expertise, and the risk of complications from high radiation doses.*

*Given Ms. Smith's medical history, a thorough evaluation of her overall health status and a discussion of the potential risks and benefits of each treatment option is recommended, with input from a multidisciplinary team of medical specialists.*

7. Mr. Miller is a 79 year-old patient with a cT2c cN0 Gleason-Score 7a prostate cancer. The disease was first diagnosed in 07/2017 after an elevated PSA of 14.2 ng/ml. His treating urologist Dr. Jones assigned him to undergo an MRI of the pelvis, where a PI-RADS 5 lesion was seen at the left-side Apex of the prostate. The lesion had contact to the capsule of the prostate, but showed no extraorganic growth or invasion of the seminal vesicles. Mr. Miller then underwent a prostate biopsy, which diagnosed an adenocarcinoma of the prostate with a Gleason score of 3+4=7a. For treatment of the cancer, the patient underwent a radical prostatectomy with pelvine lymphonodectomy in 09/2017. The final pathological result was cT2c cN0 (0/23) LVI1 Pn1 R0, Gleason 7. Mr. Miller had the following PSA values in the next couple of months: 11/17 PSA >0.1ng/ml, 01/18 PSA >0.1ng/ml, 04/18 PSA 0.1 ng/ml 07/18 0.3 ng/ml. Due to the again rising PSA value, the patient had a PSMA PET-CT, which showed no sign of tumor recurrence. Mr. Miller refused to undergo an active treatment at that moment. Further PSA values were 0.4 ng/ml in 09/18 and 0.7 ng/ml in 12/18. The PSMA-PET-CT was repeated and then showed a local uptake of the tracer near an operation clip in the lower pelvis. After the case had been discussed at the local urological tumor board, Mr. Miller was recommended to undergo salvage radiotherapy. The treatment started in 02/19 with 36 fractions up to a total dose of 72Gy of the tumor lesion and 66Gy on the prostate bed. Unfortunately, Mr. Miller suffered from diarrhea, which continued for several weeks after the treatment was finished. Furthermore, he suffered from chronic rectal bleeding. A treatment with steroids and medication for stool regulation only barely improved the symptoms. Since the radiotherapy had been done, the PSA value declined again and is on a level <0.1 ng/ml since 04/20. Mr. Miller has been in continuous follow-up ever since.

Summarize the patient history.

*Mr. Miller is a 79-year-old patient who was diagnosed with cT2c cN0 Gleason-Score 7a prostate cancer in 07/2017 after an elevated PSA level. He underwent an MRI, prostate biopsy, and radical prostatectomy with pelvine lymphonodectomy. Despite declining PSA values after the surgery, his PSA levels increased again and a PSMA-PET-CT showed a local uptake of the tracer near an operation clip. He underwent salvage radiotherapy in 02/2019, but suffered from diarrhea and chronic rectal bleeding as side effects. Since then, his PSA levels have declined and have been continuously monitored.*

8. A patient with early-stage lung cancer in the left lower lobe is planned to undergo stereotactic radiotherapy. Unfortunately, the tumor moves a lot during the breathing cycle. What could be done during the planning CT to address this problem?

*There are several techniques that can be used to address tumor motion during the planning CT for stereotactic radiotherapy of lung cancer:*

1. *4D-CT scans: This involves acquiring multiple CT scans during the breathing cycle to create a 4-dimensional (4D) image, which can then be used to visualize tumor motion and plan the radiotherapy accordingly.*
2. *Breath-hold techniques: The patient can be instructed to hold their breath at a specific point in the breathing cycle during the CT scan, which can reduce the tumor motion and improve image quality.*
3. *Respiratory-gated CT scans: This involves synchronizing the CT scan with the patient's breathing cycle to capture the images during specific phases of respiration.*
4. *Intrafractional tracking: This involves tracking the tumor position during the radiotherapy treatment to ensure that the radiation is delivered to the correct location, even if the tumor moves.*

*The most appropriate technique will depend on the patient's individual circumstances, including the severity of the tumor motion and the type of radiotherapy being planned.*

9. A 9-year-old patient with locally advanced retinoblastoma is planned to undergo radiotherapy. Generally speaking, which is the preferred radiotherapeutic modality in situations like these? Explain why.

*In the case of locally advanced retinoblastoma in a 9-year-old patient, external beam radiotherapy is generally the preferred radiotherapeutic modality. This is because it can deliver high doses of radiation to the affected eye while minimizing exposure to surrounding healthy tissues. The radiation can be targeted precisely, with the use of modern imaging and planning techniques, to ensure that the cancerous tissue receives the maximum dose while healthy tissue is spared. This approach can be effective in reducing the size of the tumor, slowing its growth, and preserving vision. Additionally, external beam radiotherapy can be given as outpatient treatment, which is convenient for both the patient and their caregivers.*

10. Make a list of possible fractionation schemes (number of fractions x dose per fraction) for a palliative analgetic radiotherapy of bone metastases.

*Here is a list of possible fractionation schemes for palliative radiotherapy of bone metastases:*

1. *8 x 3 Gy*
2. *10 x 2 Gy*
3. *5 x 4 Gy*
4. *6 x 5 Gy*
5. *20 x 1 Gy*

*It is important to note that the most appropriate fractionation scheme will depend on the individual patient's medical history, overall health, and the specific characteristics of their bone metastases. A radiation oncologist would make the final determination after careful consideration of all factors.*

11. Provide a list of dose constraints [dose/volume] for the brain stem for fractionated stereotactic radiotherapy (5 fractions) of a brain metastasis

*There is no one-size-fits-all answer to this question, as the appropriate dose constraints for the brain stem during fractionated stereotactic radiotherapy for a brain metastasis will depend on a number of factors, including the size and location of the metastasis, the patient's overall health, and the preferences of the treating physician. However, some general dose constraints for the brain stem that are often used in this setting include:*

1. *Maximum Dose: 54-60 Gy in 5 fractions*
2. *Maximum Dose per fraction: 12-13 Gy*
3. *Volume receiving at least 50% of the prescribed dose (V50): < 2 cc*
4. *Volume receiving at least 70% of the prescribed dose (V70): < 1 cc*

*It's important to note that these are just general guidelines, and the specific constraints used in each individual case may differ based on the patient's unique situation.*

12. Provide a list of dose constraints [dose/volume] for the ipsilateral lung for curative-intent radiotherapy of non-small cell lung cancer.

*The following are commonly used dose constraints for the ipsilateral lung in curative-intent radiotherapy for non-small cell lung cancer:*

1. *Maximum dose: 33 to 36 Gy*
2. *Mean lung dose: 20 to 26 Gy*
3. *V20 (volume of lung receiving at least 20 Gy): Less than 30%*
4. *V5 (volume of lung receiving at least 5 Gy): Less than 50%*
5. *Maximum point dose: No constraint or up to 60 Gy*

*It's important to note that the dose constraints may vary based on individual patient factors, the radiotherapy technique used, and institutional preferences. A radiation oncologist should be consulted for specific recommendations.*

13. Describe how Level I-II of the axilla may be contoured for radiation treatment planning of nodal positive breast cancer; mention anatomical borders for orientation.

*In radiation treatment planning of nodal positive breast cancer, Level I-II of the axilla may be contoured to target the lymph nodes in this area.*

*Anatomical borders for orientation of the axilla include:*

- *Medial border: the pectoralis muscle*
- *Lateral border: the humerus bone*
- *Superior border: the axillary vein*
- *Inferior border: the axillary fold*

*Level I-II of the axilla should be contoured to include the lymph nodes in this region, with attention to the interpectoral (Rotter's) node, which is the most important node in the axilla. The level I-II nodes should be included from the lateral border of the pectoralis muscle to the lateral border of the humerus.*

*It is important to note that the axilla should be contoured based on the specific patient's anatomy, as individual variations may exist.*

14. A patient with glioblastoma in the right temporal lobe is planned to undergo radiotherapy. What are important organs at risk that should be delineated for contouring?

*During radiotherapy for a glioblastoma in the right temporal lobe, it is important to delineate and contour the following organs at risk (OARs) to minimize potential radiation toxicity:*

1. *Optic nerves and chiasm - to preserve vision*
2. *Brainstem - to preserve vital functions such as breathing, swallowing and consciousness*
3. *Pituitary gland - to preserve endocrine function*
4. *Temporal and frontal lobes - to minimize cognitive and memory deficits*
5. *Inner ear - to preserve hearing*
6. *Spinal cord - to minimize myelitis*
7. *Parotid glands - to preserve saliva production*

*It is important to properly contour these OARs to ensure safe and effective radiotherapy delivery while minimizing toxicity to normal surrounding tissues.*

15. A patient with lymph node positive squamous cell carcinoma of the palatine tonsil is planned to undergo radiotherapy up to 32x2Gy=64Gy to the high-risk volume. The calculation of the radiation treatment planning led to the following dose parameters:

-Parotid gland left: Mean Dose 20 Gy, Max Dose 65 Gy

-Parotid gland right: Mean Dose 5 Gy, Max Dose 10 Gy

-Brain Stem: Max Dose 40 Gy

Please evaluate the acceptability of these dose parameters.

*The dose parameters appear to be within the accepted limits for radiotherapy treatment of squamous cell carcinoma of the palatine tonsil.*

*The mean dose of 20 Gy and max dose of 65 Gy to the left parotid gland and mean dose of 5 Gy and max dose of 10 Gy to the right parotid gland are generally considered acceptable, as long as they are not exceeding the established tolerance limits for these organs.*

*The max dose of 40 Gy to the brain stem is also considered acceptable, as it is below the established tolerance limit.*

*It is important to note that these dose parameters may not be applicable for every individual, as every case is unique and other factors such as patient anatomy, treatment goals, and overall health must be taken into account.*

*In conclusion, these dose parameters appear to be acceptable, but a thorough evaluation and discussion with the treating physicians is necessary to confirm their suitability for the specific case.*

16. A patient with intermediate-risk prostate cancer is planned to undergo radiotherapy with a fractionation scheme of 20x3Gy=60Gy. The calculation of the radiation treatment planning led to the following dose parameters:

-Bladder: V40Gy=80%, V50Gy=60%, V60Gy=55%, Dmax=67Gy

-Rectum: V40Gy=70%, V50Gy=60%, V60Gy=50%, Dmax=65Gy

Please evaluate the acceptability of these dose parameters.

*The dose parameters listed for the bladder (V40Gy=80%, V50Gy=60%, V60Gy=55%, Dmax=67Gy) and rectum (V40Gy=70%, V50Gy=60%, V60Gy=50%, Dmax=65Gy) are commonly used as indicators of normal tissue toxicity in prostate cancer radiotherapy. A higher volume of tissue receiving a higher dose (V40Gy, V50Gy, V60Gy) increases the risk of normal tissue toxicity, while a higher maximum dose (Dmax) also increases the risk of toxicity.*

*In general, a V40Gy of less than 85%, V50Gy of less than 65% and V60Gy of less than 60% are considered acceptable. A Dmax less than 70Gy is also considered acceptable. Based on these standards, the dose parameters for the bladder and rectum appear to be within acceptable limits. However, it's important to keep in mind that each patient's individual situation may require a different approach and these values are only a general guideline.*

*In conclusion, the dose parameters listed for the bladder and rectum are generally considered acceptable, but a thorough evaluation of the patient's individual medical history and conditions is necessary to determine the best plan for their specific case.*

17. What is 20x3Gy in EQD2 based on the linear-quadratic model using an α/β=2?

*The equivalent dose in 2 Gy fractions (EQD2) can be calculated using the linear-quadratic model and an α/β of 2 as follows:*

*EQD2 = 20 x 3Gy * (2 + (2/α/β)) = 20 x 3Gy * (2 + (2/2)) = 20 x 3Gy * 4 = 240 Gy*

*So, the EQD2 for 20 fractions of 3 Gy each with an α/β of 2 is 240 Gy.*

18. For palliative radiation therapy, a fractionation scheme of 10x3Gy is used. Based on the linear-quadratic model using an α/β=2, which dose per fraction would be needed for a biologically equivalent dose in 5 fractions?

*The biologically equivalent dose in 5 fractions would be 15 Gy. This can be calculated using the linear-quadratic model with an α/β of 2:*

*Dequivalent = (10 Gy / fraction) * (1 + (2 * 2 * 3Gy/fraction)) = 15 Gy.*

19. A patient with metastatic renal cell carcinoma is under radiotherapy for palliative analgesic irradiation of a bone metastasis in the lumbal spine. Unfortunately, the execution of the therapy is impaired, because it is difficult for the patient to lie still due to his pain. Make some suggestions, what may be done to overcome this problem.

*There are several steps that may be taken to overcome the difficulty of a patient lying still during radiotherapy for a bone metastasis in the lumbar spine:*

1. *Pain management: The patient's pain should be managed with medication and other modalities to ensure they are as comfortable as possible during the therapy.*
2. *Positioning aids: The patient may be positioned using pillows, cushions, or other aids to help them maintain a comfortable and stable position during the therapy.*
3. *Anesthesia: If necessary, the patient may be given a mild anesthesia to help them relax and remain still during the therapy.*
4. *Sedation: In some cases, patients may require sedation to help them relax and remain still during the therapy.*
5. *Image guidance: Image guidance techniques, such as X-rays or other imaging modalities, may be used to help the patient remain in the correct position during the therapy.*
6. *Radiation shielding: Shielding may be used to protect sensitive areas and prevent movements during the therapy.*
7. *Interventional procedures: In some cases, interventional procedures, such as vertebroplasty or kyphoplasty, may be performed prior to the radiotherapy to help reduce pain and improve the patient's ability to lie still.*

20. Provide a list of the most important side effects of a percutaneous radiotherapy of the breast a patient should know about.

*Some common side effects of percutaneous radiotherapy of the breast include:*

1. *Skin irritation or redness at the site of treatment*
2. *Fatigue*
3. *Swelling and tenderness in the breast*
4. *Nausea and vomiting*
5. *Diarrhea*
6. *Loss of appetite*
7. *Pain or discomfort in the breast or chest*
8. *Shortness of breath*
9. *Skin changes, such as darkening or dryness*
10. *Muscle or joint pain*

*It is important to note that these side effects may vary from patient to patient and may not occur in all cases. It is recommended to discuss possible side effects with your treating doctor.*

21. A patient with limited disease small-cell lung cancer had chemotherapy with concurrent radiotherapy for 6 weeks. After 4 weeks of the treatment he is beginning to develop symptoms of pain during swallowing (VAS 6/10), which he describes as burning. He also suffers from impaired swallowing, which is why he reduced eating. What is the most likely reason for the symptoms and how could you help the patient?

*The most likely reason for the patient's symptoms of pain during swallowing and impaired swallowing is radiation-induced esophagitis, which is a common side effect of concurrent chemoradiotherapy for lung cancer. The symptoms can be due to inflammation and injury of the esophageal mucosa caused by the radiation.*

*To help the patient, the following measures can be taken:*

1. *Pain management: Over-the-counter pain relievers or prescription pain medication can be used to manage the pain.*
2. *Diet modification: Soft, bland foods and frequent small meals can help alleviate symptoms and improve swallowing. Avoiding spicy and acidic foods can also help.*
3. *Mouth care: Good oral hygiene, such as regular mouth rinses and gargles, can help to soothe the throat and prevent infections.*
4. *Lubricating agents: Using agents such as honey, glycerin, or saliva substitutes can help to lubricate the throat and improve swallowing.*

*It is important to consult a medical professional for proper evaluation and management of the patient's symptoms, as the above measures may not be sufficient to alleviate symptoms in all cases. Additionally, if the symptoms persist or worsen, further investigations such as endoscopy may be necessary to rule out other causes.*

22. Write a physician letter. The date of the letter is 12^th^ of June 2022. The treatment is already over; include only the medical information relevant for the radiotherapy. The data is the following:

From: Dr. Ray Oncology (radiation oncologist)

To: Dr. Michael Hoover (general doctor), address: Winter road 85, 12345 Example Town

Name: Lisa Ramsey

Age: 85

Diagnosis: Basalioma on the nose

Further diagnoses:

-M. Meniére

-Diabetes mellitus type 2

-Operation for Hip prothesis (left) in 2014

Treatment details:

Start of Treatment: 9^th^ of May 2022

End of Treatment: 27^th^ of May 2022

Prescription dose: 12 x 4 Gy

Radiation type: Photons

Radiation technique: IMRT (VMAT)

Positioning devices: radiation mask, bolus

Side effects: Radiodermatitis grade I with reddening of the skin; treated with local skin cream, no serious side effects

Notes: Operation was refused by the patient. Patient lives in nursery home (Address: Summer Road 92 in 2374 Sample Town)

*Dear Dr. Hoover,*

*I am writing to update you on the medical information of your patient, Lisa Ramsey, who underwent radiation therapy for the treatment of Basalioma on the nose.*

*Ms. Ramsey, aged 85, was diagnosed with Basalioma on the nose and had a history of M. Meniére, Diabetes Mellitus type 2, and a previous operation for Hip prothesis (left) in 2014. The treatment started on the 9th of May 2022 and ended on the 27th of May 2022. During the treatment, Ms. Ramsey received 12 fractions of 4 Gy of Photons delivered through IMRT (VMAT) technique, using a radiation mask and bolus as positioning devices.*

*During the course of treatment, the patient developed Radiodermatitis grade I with reddening of the skin, which was treated with local skin cream. No serious side effects were reported. An operation was refused by the patient.*

*Please note that Ms. Ramsey lives in a nursing home at Summer Road 92 in 2374 Sample Town.*

*Please do not hesitate to contact me if you have any further questions or concerns.*

*Sincerely, Dr. Ray Oncology Radiation Oncologist*

23. Summarize the main findings of the PORTEC III trial

*The PORTEC III trial (Postoperative Radiation Therapy in Endometrial Carcinoma III) was a clinical trial aimed at evaluating the effectiveness of postoperative radiation therapy in women with endometrial cancer. The main findings of the trial showed that postoperative radiation therapy significantly improved disease-free survival and reduced the risk of cancer recurrence, particularly in women with high-risk features. The study concluded that the use of adjuvant radiation therapy should be considered in women with endometrial cancer who are at high risk of recurrence.*

24. Summarize the main findings of the QUARTZ trial, which investigated whole brain radiotherapy in patients with brain metastases.

*The QUARTZ trial investigated the efficacy and safety of whole brain radiotherapy (WBRT) in patients with brain metastases. The trial found that WBRT combined with cognitive rehabilitation did not result in a significant improvement in cognitive function compared to cognitive rehabilitation alone. However, WBRT was found to be associated with a lower risk of symptomatic brain progression compared to observation alone. These findings suggest that WBRT may still have a role in the management of brain metastases, particularly in patients with significant symptoms, but it should be used with caution given its potential impact on cognitive function.*

25. What is the rationale behind hyperthermia in radiotherapy from a radiobiological point of view?

*Hyperthermia in radiotherapy is a radiobiological technique that involves the use of heat to enhance the effects of ionizing radiation on cancer cells. This approach is based on the idea that cancer cells are more sensitive to damage from heat than normal cells, making them more susceptible to the effects of radiation therapy. By raising the temperature of the tumor tissue, hyperthermia can increase the effectiveness of radiation therapy by increasing the amount of damage inflicted on cancer cells and reducing the damage to normal tissues. Additionally, hyperthermia has been shown to increase the activity of certain enzymes involved in DNA repair, which can further enhance the effects of radiation therapy.*
