# Supplementary material for: Exploring Capabilities of Large Language Models such as ChatGPT in Radiation Oncology
Source: Adv Radiat Oncol. 2023 Nov 4;9(3):101400. doi: 10.1016/j.adro.2023.101400 (PMC10831180; doi:10.1016/j.adro.2023.101400)
Supplement: Appendix3 - Additional Figures [file mmc3.docx]

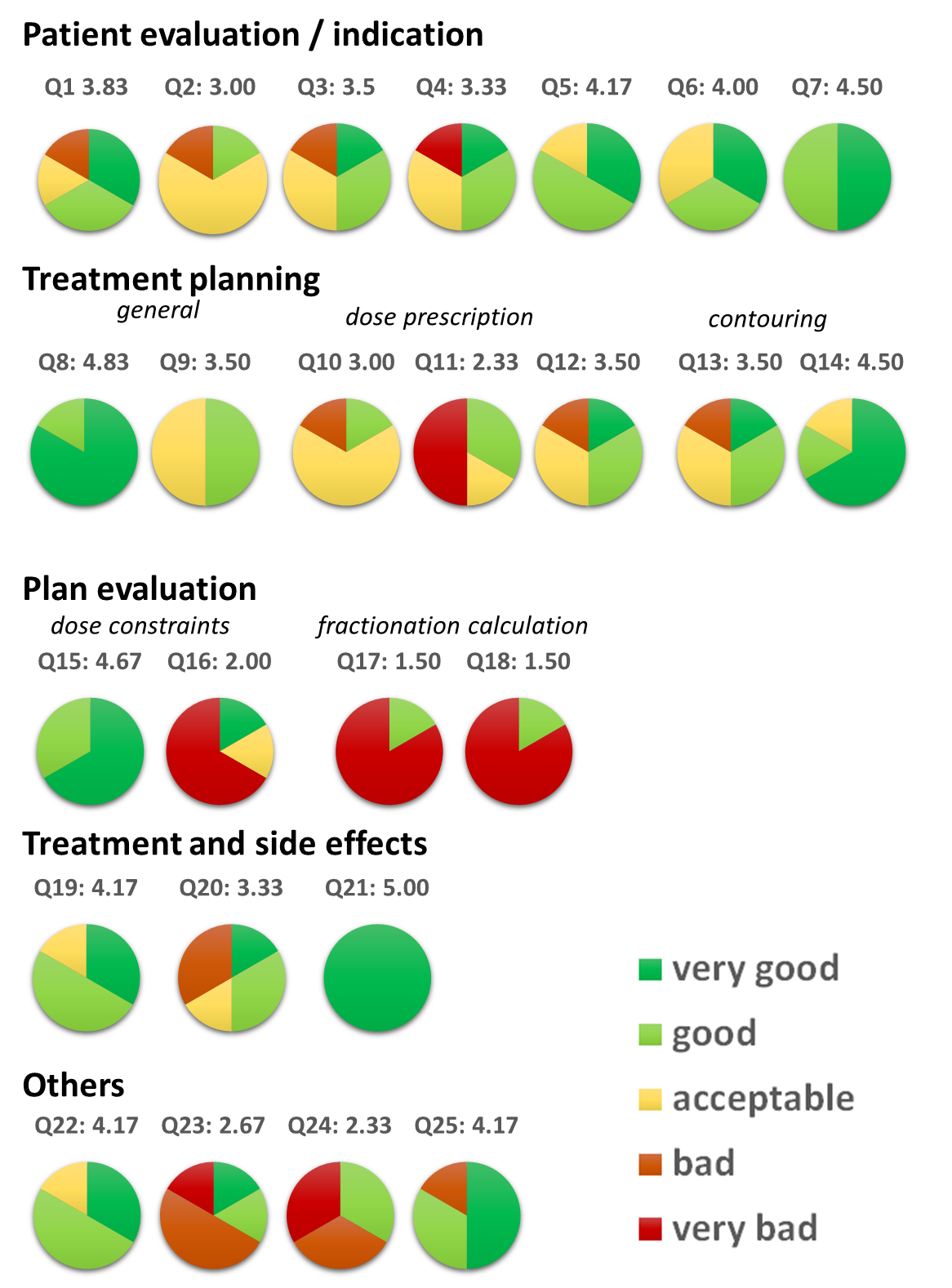


Fig. E1: Evaluation results regarding the usefulness of the answers given by ChatGPT. Score values of each answer are calculated as mean of individual score values given by the radiation oncologists.
