# Supplementary material for: Exploring Capabilities of Large Language Models such as ChatGPT in Radiation Oncology
Source: Adv Radiat Oncol. 2023 Nov 4;9(3):101400. doi: 10.1016/j.adro.2023.101400 (PMC10831180; doi:10.1016/j.adro.2023.101400)
Supplement: Appendix4 - Additional Tables [file mmc4.docx]

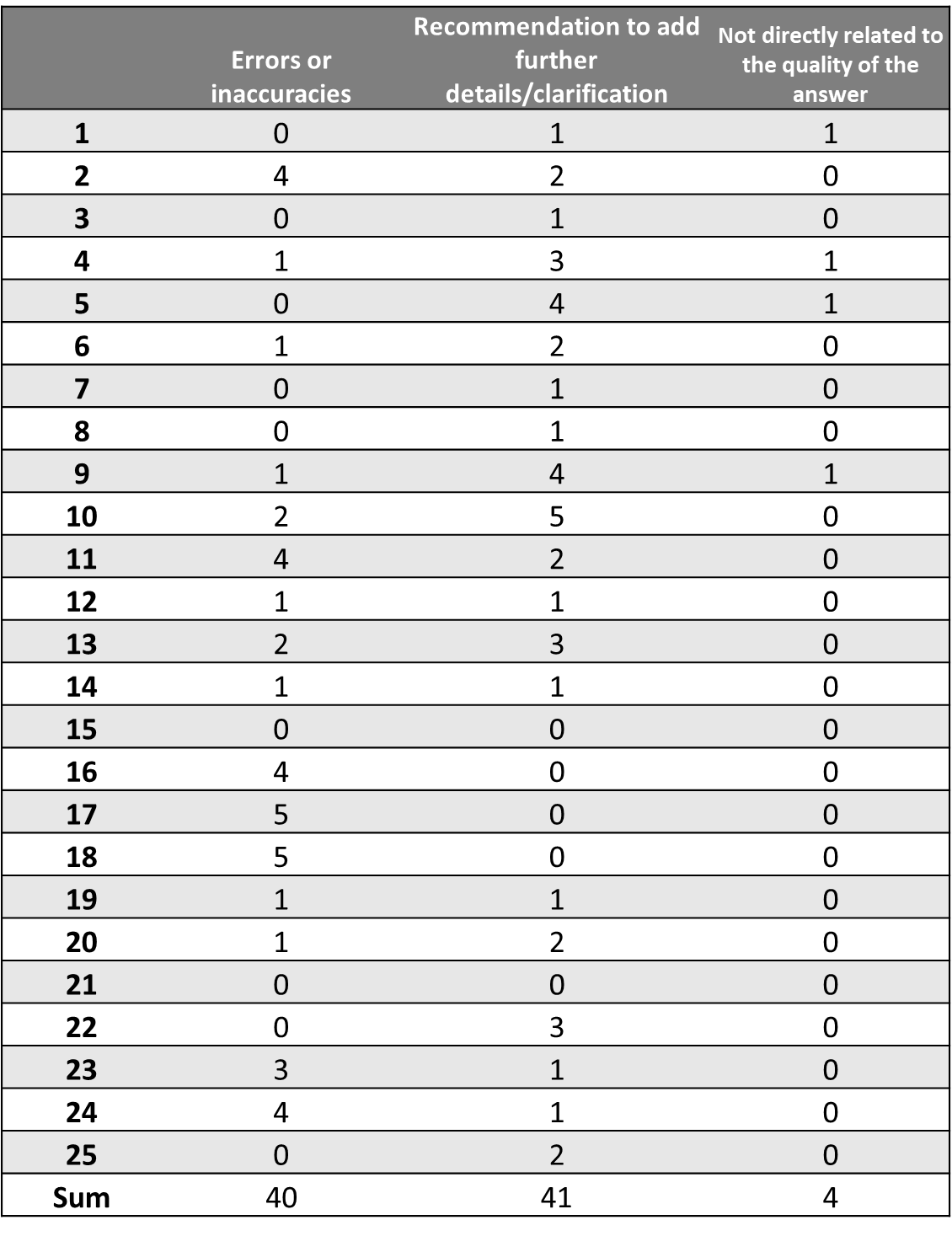


Table E1: Results of the content-analysis by classification of the comments given by the radiation oncologists.


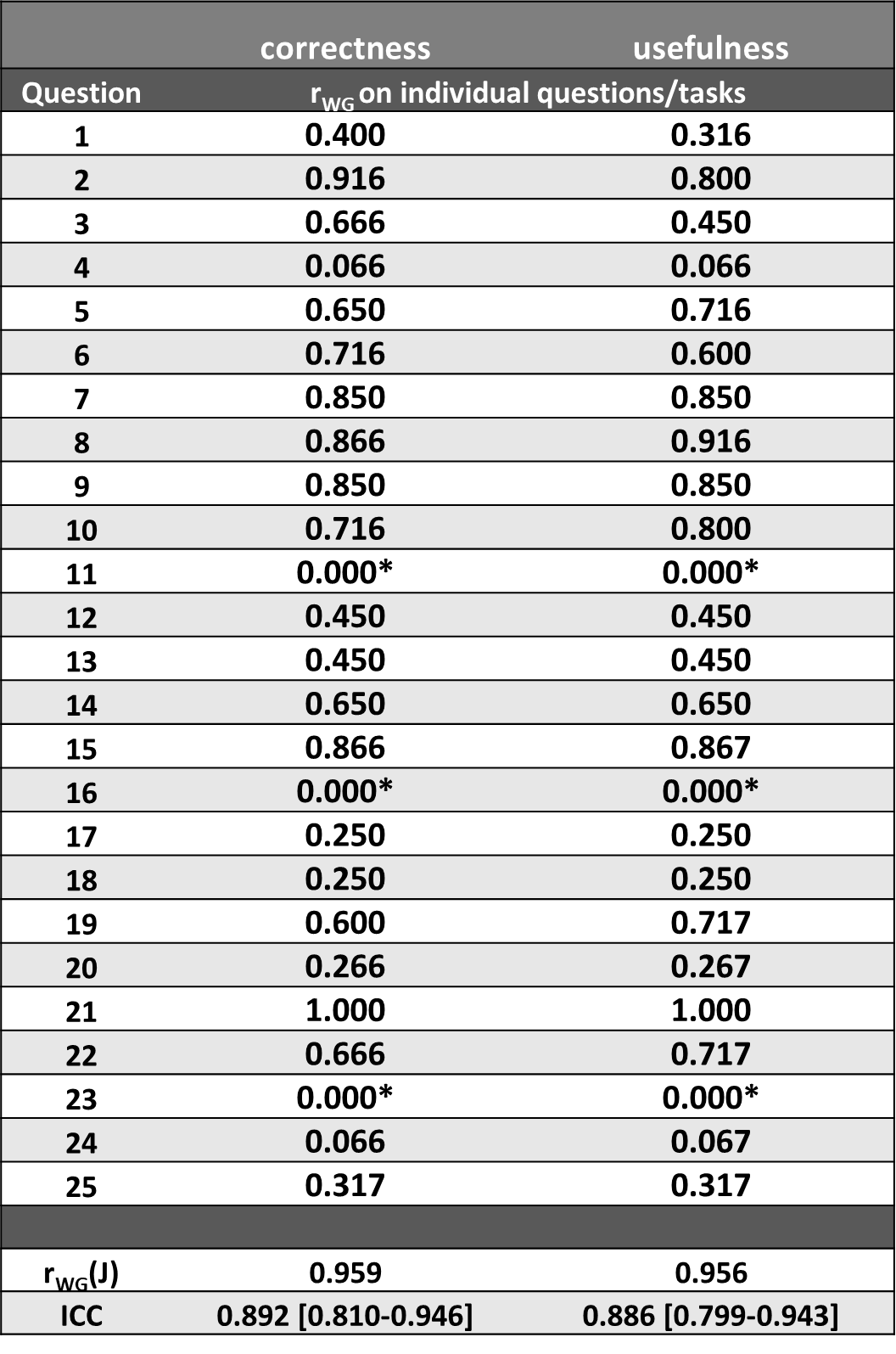


Table E2: Results on interrater agreement by calculation rWG for individual questions/tasks, as well as rWG(J) and ICC for overall agreement. *values below 0 are reset to 0.
